# Supplementary material for: Inhibition of Hepatitis E Virus Replication by Novel Inhibitor Targeting Methyltransferase
Source: Viruses. 2022 Aug 15;14(8):1778. doi: 10.3390/v14081778 (PMC9415367; doi:10.3390/v14081778)
Supplement: Supplementary file 1 [file viruses-14-01778-s001.zip › viruses-1828368-supplementary.pdf]

Inhibition of Hepatitis E Virus Replication by novel inhibitor targeting Methyltransferase

Preeti Hooda[1], Meenakshi Chaudhary[1], Mohammad K. Parvez[2*], Neha Sinha[3] and Deepak Sehgal[1]*

[1]Virology Laboratory, Department of Life Sciences, Shiv Nadar University, Gautam Budh Nagar, Greater Noida, India.

[2]Department of Pharmacognosy, College of Pharmacy, King Saud University, Riyadh, Saudi Arabia.

[3]Department of Infectious Diseases and Microbiology, School of Public Health, University of Pittsburgh, Pittsburgh, PA, USA

*Correspondence: Prof Deepak Sehgal, Professor, Department of Life Sciences, Shiv Nadar University, Gautam Budh Nagar, 201314, Greater Noida, India, deepak.sehgal@snu.edu.in

*Co-correspondence: Mohammad K. Parvez, Professor, Department of Pharmacognosy, College of Pharmacy, King Saud University, Riyadh, Saudi Arabia. mohkhalid@ksu.edu.sa

Supplementary material:

A comprehensive computational investigation of the HEV has been performed for Methyltransferase (MTase) using molecular docking approaches to observe significant similarities with other viral MTases. Blast results showed that the protein appears in the twilight zone of protein modelling, showing the amino acid sequence alignment of *Human* (PDB ID: 3BGV) and HEV-MTase as 18% identity and 30% similarity. The values observed are less than 20% when comparing per cent identity amongst the sequences. When nsp1 (*Semliki Forest virus* (SFV)) was compared to pORF1 (HEV MTase), 19% identity and 31% similarity were observed **(Figure S1).** When other virus MTases, such as *Dengue virus* (PDB: 2P1D), compared to HEV, 16 % identities and 30% similarities were observed. When nsp1(*Chikungunya* MTase) was compared to HEV MTase, 20 % identity and 34% similarity were observed.

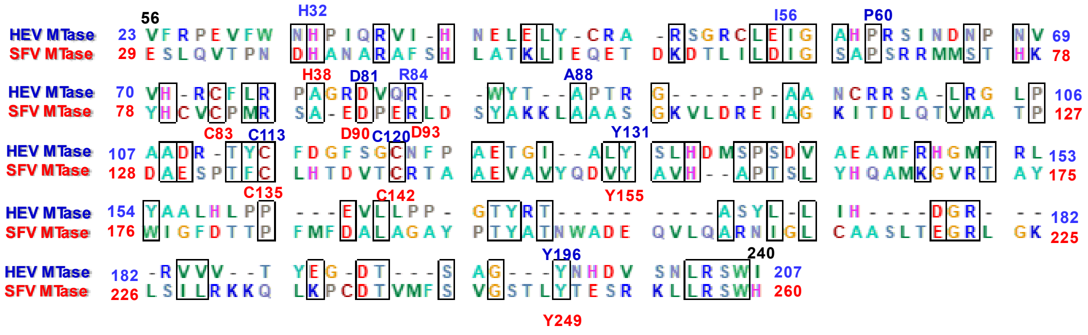

Figure S1: Amino acid Sequence alignment of HEV-MTase (23 to 207 correspond to (Putative HEV-MTase 56 to 240 aa region) and SFV-MTase domain 29 to 260 region. Conserved residues of HEV and SFV are highlighted in blue and red colours, respectively.

Molecular modeling of HEV-MTase

Homology modelling validation and refinement of HEV-MTase model

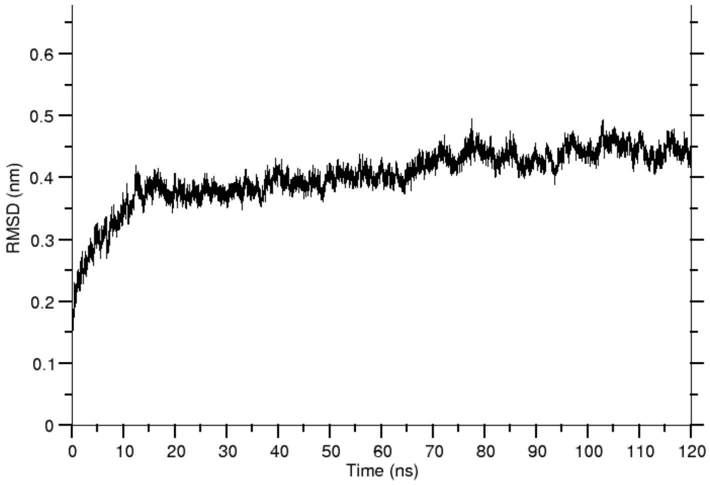

Figure S2: Refinement of HEV-MTase structure model by MD simulations. RMSD plot of trajectories from 120 ns MD simulations.

HEV MTase Model validation

The refined 3D model was validated based on stereochemical and geometric considerations. The top-ranked model was further validated and analyzed based on their Ramachandran plot and ProSA-web programs **(Figure S3)** [54,55]. The refined HEV-MTase structure was used for further docking and all other analyses in the present study.

The stereochemical quality of the modelled structure increased considerably by MD simulation. In contrast, the residue count increased significantly in the favoured and allowed region (95.6% -96.3%) and decreased in the outlier (2.2% - 1.8 %) as well as permitting regions (from 2.2% to 1.8%) **(Figure S3.A, B** and **Table S1).**

Furthermore, validation of the refined structure model, based on z-score (for overall model quality) and energy of residues (for local model quality), indicates that the model was of good quality and energetically stable. The Z-score of the refined model was -6.23 (in the range of z-score for PDB structures determined by NMR) **(Figure S3.C)**. Analysis of the 3D model based on residue energies indicated that most residues were in a low energy state (**Figure S3.D)**. Energy versus sequence length plots for a window size of 10 and 40 amino acids further validated its stability **(Figure S3.E)**.

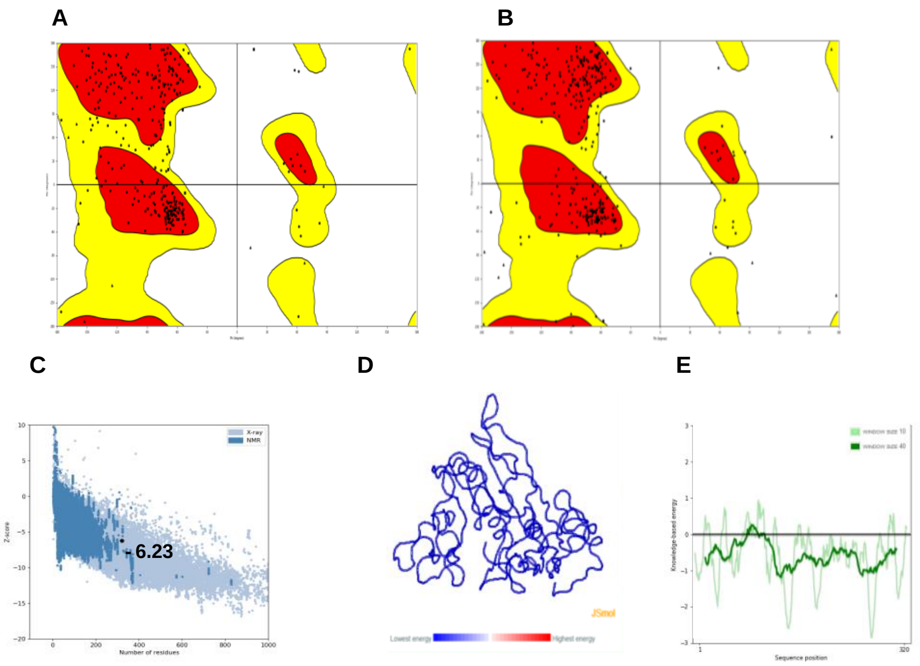

Figure S3. Validation of predicted 3D structure model of HEV-MTase protein. A. Ramachandran plot before MD simulations; B. Ramachandran plot after 110 ns MD simulations; C. Z-plot of the refined 3D model. The number written on the plot represents the z-score value of the model; D, 3D model showing its energy by BWR colour scheme, the transition from blue to red colour of model shows increase in energy; E. Energy vs. sequence position plot showing energies as a function of amino acid sequence position, light green and dark green lines on the plot represent energies over a range of 10 and 40 amino acids respectively.

Table S1: Number of residues in favoured, allowed, and outlier regions before and after MD simulations

	**Before MD simulation**		**After MD simulation**	
	**Residue**	**%**	**Residue**	**%**
No. of residues in favoured region	198	73.1	207	76.4
No. of residues in the allowed region	61	22.5	54	19.9
No. of residues in the generously allowed region	6	2.2	5	1.8
No. of residues in outlier region	6	2.2	5	1.8
Number of non-glycine and non-proline residues	271		271	
Number of end-residues (excl. Gly and Pro)	2		2	
Number of glycine residues (shown as triangles)	20		20	
Number of proline residues	27		27	
Total number of residues	320	100	320	100

Table S2: Drug-like properties of selected compounds

Compound Number	MW	QPpolrz	QPlogPC16	QPlogPoct	QPlogPw	QPlogPo/w	QPlogS	QPlogHERG	QPPCaco	QPlogBB	QPPMDCK	QPlogKp	%HumanOralAbsorption
Compound 1	166	17	6	10	8	-1	-2	-2	74	-1	37	-4	68
Compound 2	152	14	6	9	8	1	-1	-1	84	-1	43	-4	68
Compound 3	357	36	13	24	19	1	-3	-6	220	-2	96	-3	72
Compound 4	285	28	10	17	14	1	-2	-4	184	-1	142	-3	71
Compound 5	313	33	11	19	16	1	-3	-4	90	-1	69	-3	67
Compound 6	194	15	6	16	16	-2	-1	-3	257	-1	114	-4	61
Compound 7	373	38	14	28	23	0	-3	-6	28	-3	10	-5	50
Compound 8	367	42	13	22	15	2	-3	-5	83	-1	62	-5	73
Compound 9	385	37	14	29	25	-1	-3	-6	50	-2	20	-4	28
Compound 10	297	32	11	18	15	1	-3	-5	92	-2	66	-3	67

Principal Descriptors Calculated for the Best Hit Compounds:

QPpolrz: Predicted polarizability in cubic angstroms. (Acceptable range: -13.0 – 70.0), QPlogPC16: Predicted hexadecane/gas partition coefficient. (Acceptable range: - 4.0 – 18.0), QPlogPoct: Predicted octanol/gas partition coefficient. (Acceptable range: 8.0 – 35.0), QPlogPw: Predicted water/gas partition coefficient. (acceptable range: - 4.0 – 45.0), QPlogo/w: Predicted octanol/water partition coefficient log p (acceptable range: -2.0 to 6.5), QPLogS: Predicted aqueous solubility; S in mol/L (acceptable range: -6.5), QPlogHERG: Predicted IC50 value for blockage of HERG K+ channels (Concern below -5.0), QPPCaco: Predicted apparent Caco-2 cell permeability in nm/sec. Caco-2 cells are a model for the gut blood barrier. QikProp predictions are for non-active transport. (<200 poor and >500 great), QPlogBB: Predicted brain/blood partition coefficient. Note: QikProp predictions are for orally delivered drugs, so, for example, dopamine and serotonin are CNS negative because they are too polar to cross the blood-brain barrier (Acceptable range –3.0 – 1.2), QPPMDCK: Predicted apparent MDCK cell permeability in nm/sec. MDCK cells are considered to be a good mimic for the blood-brain barrier. QikProp predictions are for

non-active transport (<25 poor and >500 great), QPlogKp: Predicted skin permeability, log Kp. (Acceptable range –8.0 to –1.0), Percentage of human oral absorption (<25% is poor and >80% is high).

Table S3: IUPAC Name of the ten compounds selected after virtual screening against SAM binding site of HEV-MTase.

Compound Number	Chemical formula	IUPAC Name
Compound 1	$C_9H_{10}O_3$	3-(4-hydroxyphenyl)propionic acid
Compound 2	$C_8H_7O_3^-$	2-(4-hydroxyphenyl)acetate
Compound 3	$C_{17}H_{19}N_5O_4$	2-[6-(benzylamino)purine-9-yl]-5-(hydroxymethyl)oxolane-3,4-diol
Compound 4	$C_{15}H_{11}NO_5$	2-{[(2Z)-2-[(furan-2-yl)methylidene]-3-oxo-2,3-dihydro-1-benzofuran-6-yl]oxy}acetamide
Compound 5	$C_{16}H_{15}N_3O_4$	2-[(3*S*)-2,5-dioxo-3,4-dihydro-1*H*-1,4-benzodiazepin-3-yl]-*N*-(furan-2-ylmethyl)acetamide
Compound 6	$C_7H_{14}O_6$	(2R,3S,4S,5S,6S)-2-(hydroxymethyl)-6-methoxyoxane-3,4,5-triol
Compound 7	$C_{17}H_{19}N_5O_5$	8-(benzylamino)-9-[3,4-dihydroxy-5-(hydroxymethyl)oxolan-2-yl]-1*H*-purine-6-one
Compound 8	$C_{20}H_{25}N_5O_2$	N-(3-oxo-3-{3,5,6,7-tetrahydrospiro[imidazo[4,5-c]pyridine-4,4'-piperidin]-1'-yl}propyl)benzamide

to 0.5),	Compound 9	$C_{17}H_{19}N_7O_4$	(2R,3R,4S,5R)-2-[(8E)-6-amino-8-[(2E)-2-(phenylmethylidene)hydrazin-1-ylidene]-8,9-dihydro-7H-purine-9-yl]-5-(hydroxymethyl)oxolane-3,4-dio
	Compound 10	$C_{14}H_{15}N_7O$	3-[(9H-purine-6-yl)amino]-N-[(pyridine-3-yl)methyl]propanamide

References:

54. Wiederstein M, Sippl MJ. ProSA-web: interactive web service for recognizing errors in three-dimensional structures of proteins. Nucleic Acids Research. 2007;35: W407–W410. doi:10.1093/nar/gkm290

55. Sippl MJ. Recognition of errors in three-dimensional structures of proteins. Proteins: Structure, Function, and Bioinformatics. 1993;17: 355–362. doi:10.1002/prot.340170404
